# Syndrome Differentiation of IgA Nephropathy Based on Clinicopathological Parameters: A Decision Tree Model

**DOI:** 10.1155/2017/2697560

**Published:** 2017-03-26

**Authors:** Yanghui Gu, Yu Wang, Chunlan Ji, Ping Fan, Zhiren He, Tao Wang, Xusheng Liu, Chuan Zou

**Affiliations:** ^1^Renal Division, Second Clinical Medical College, Guangzhou University of Traditional Chinese Medicine, Guangzhou 510006, China; ^2^Renal Division, Shanxi Provincial Hospital of Chinese Medicine, Xi'an 710200, China; ^3^Renal Division, Guangdong Provincial Hospital of Chinese Medicine, Guangzhou 510120, China; ^4^Renal Division, Peking University Third Hospital, Beijing 100191, China

## Abstract

*Background.* IgA nephropathy is the most common cause of primary glomerulonephritis in China, and Traditional Chinese Medicine (TCM) is a vital treatment strategy. However, not all doctors prescribing TCM medicine have adequate knowledge to classify the syndrome accurately.* Aim.* To explore the feasibility of differentiation of TCM syndrome types among IgA nephropathy patients based on clinicopathological parameters.* Materials and Methods.* The cross-sectional study enrolled 464 biopsy-proven IgA nephropathy adult patients from 2010 to 2016. The demographic data, clinicopathological features, and TCM syndrome types were collected, and the decision tree models based on classification and regression tree were built to differentiate between the syndrome types.* Results.* 370 patients of training dataset were 32 years old with serum creatinine of 79 *μ*mol/L, estimated glomerular filtration rate (eGFR) of 97.2 mL/min/1.73 m^2^, and proteinuria of 1.0 g/day. The scores of Oxford classifications were as follows: M1 = 97.6%, E1 = 14.6%, S1 = 50.0%, and T1 = 52.2%/T2 = 18.4%. The decision trees without or with MEST scores achieved equal precision in training data. However, the tree with MEST scores performed better in validation dataset, especially in classifying the syndrome of qi deficiency of spleen and kidney.* Conclusion.* A feasible method to deduce TCM syndromes of IgA nephropathy patients by common parameters in routine clinical practice was proposed. The MEST scores helped in the differentiation of TCM syndromes with clinical data.

## 1. Introduction

IgA nephropathy has been recognized as the most common form of primary glomerulonephritis [1–5] and is a leading cause of chronic kidney disease (CKD) and end-stage renal disease (ESRD) [6]. In China, IgA nephropathy contributed to 32–54% of primary glomerulonephritis [3, 7], and >30% of IgA nephropathy patients would progress to ESRD within 20 years after biopsy [7]. Although recommended by the guidelines, steroids and immunosuppressive agents are not suitable for all patients due to side effects [8], especially for those with their estimated glomerular filtration rate (eGFR) less than 30 mL/min/1.73 m^2^. Similar to several other developing countries, alternative therapies are considered as pivotal and general treatment strategies for IgA nephropathy in China, especially the decoction and the patent medicine of TCM [9–11]. In fact, IgA nephropathy patients have benefited from TCM treatment, and the mechanism has been partially unveiled [12–15].

The experience of 3000 years and modern researches show that Chinese Medicine must be applied with syndrome classification under the direction of TCM theory. Only in this way could the effectiveness be significant and the adverse events be avoided [16]. Syndrome is mainly identified by elements containing rich information of TCM, including medical history, symptoms, and signs, which looks a little like symptom cluster. The elements are commonly collected by observation, listening/smelling, questioning, and pulse analyses. Western medicine and TCM have different exploring dimensions but share the same study objects. In recent years, continued in-depth study has got close connection between modern disease and TCM in multiple levels [17], such as the syndromes and clinicopathological parameters in IgA nephropathy [18]. Thus, TCM syndromes have shortcomings in objectivity and consistency and are hard to be spread, especially to Western doctors. Is there any method to support those clinicians with little TCM knowledge in improving the ability of syndrome differentiation? The TCM syndrome types have been reported to demonstrate various clinical and pathological features [19–22]. The method in the present study described a decision tree as a predictive model, which maps observations to deduce the target value of an element in detail [23, 24]. The observations are shown on the branches of the tree and the target is represented in the leaves. During analysis, a decision tree can be used to visually and explicitly account for the process of decision-making, which makes it one of the predictive modeling approaches most commonly used in statistics, data mining, and machine learning. Therefore, we attempted to use the decision tree in order to explore the feasibility of syndrome differentiation among IgA nephropathy patients based on clinicopathological parameters.

## 2. Materials and Methods

### 2.1. Patients

370 biopsy-proven IgA nephropathy patients from 1 January 2010 to 31 December 2014, in Guangdong Provincial Hospital of Chinese Medicine and Shanxi Provincial Hospital of Chinese Medicine, and 94 from 1 January 2015 to 30 June 2016, in Guangdong Provincial Hospital of Chinese Medicine, were enrolled in the present study after excluding patients below 14 years of age and those with Henoch-Schönlein purpura, systemic lupus erythematosus, and cirrhosis. The patients were categorized into two sets: training dataset and validation dataset. The training dataset included 370 patients during 2010–2014, while the validation dataset consisted of 94 patients during 2015-2016.

### 2.2. Pathological Studies

The standard process of interpreting renopuncture tissues included light microscopy, immunofluorescence, and electron microscopy. For light microscopy, all specimens were stained with hematoxylin and eosin (H&E), Periodic acid–Schiff, Masson's trichrome, and Jones' methenamine silver. Renal biopsies were scored according to the Oxford MEST scoring system [25] by two pathologists who were blinded to the patients' type of syndromes. An agreement on the definitions and scoring of pathological features was essential during the pathological review.

### 2.3. Syndrome Differentiation

The evaluation of TCM syndromes was performed according to the Guiding Principle of Clinical Research on New Drugs of Traditional Chinese Medicine [26] for the treatment of chronic nephropathy. The information on the syndromes was acquired from the record of latest ward round by the Chinese medical practitioner. In the training dataset, 273 patients showed qi deficiency of spleen and kidney (QDSK), 66 were with both qi and yin (DBQY), 19 exhibited yang deficiency of spleen and kidney, 8 got yin deficiency of liver and kidney, 3 presented lower energizer damp-heat, and 1 had lung wind-heat. To increase the power of statistics, the first four syndrome types with smallest sample sizes were combined in a category of other types (OTs). In the validation dataset, 80 patients acquired QDSK and 14 acquired DBQY.

### 2.4. Statistical Analysis

Continuous data with normal distribution were presented as the mean and standard deviation (SD), and those with abnormal distribution were presented as a median and interquartile range. The ranked data with equal intervals were expressed as median and interquartile range, and those with unequal intervals were expressed as counts and percentages of each rank. The categorical variables were expressed as counts and percentages. Comparisons between groups were conducted using independent-samples *t*-test or Kruskal-Wallis test for continuous variables and chi-squared test or Fisher's exact test for categorical variables. All the mentioned tests were two-tailed, and a *P* value < 0.05 was considered statistically significant. The analyses were conducted by SPSS 17.0 statistics software (SPSS, Inc., Chicago, IL). The decision tree was built by R with packages of rpart and rpart.plot based on the data from 390 patients biopsied between 1 January 2010 and 31 December 2014. Variables involved in modeling included gender, age, mean arterial pressure, disease course, macroscopic hematuria or not, eGFR, uric acid, serum albumin, hemoglobin, proteinuria, and urinary red blood cell. The MEST scores were added to the model to explore whether pathological parameters would increase the accuracy of the model. Positive predictive value (PPV), negative predictive value (NPV), integrated discrimination improvement (IDI), and net reclassification index (NRI) were calculated to evaluate the improvement in predicting QDSK of the model with MEST scores.

## 3. Result

### 3.1. Demographic and Clinical Data of Training Dataset

Table 1 lists the demographic and clinical characteristics of biopsy of the training dataset with IgA nephropathy, and the clinicopathological parameters of different TCM syndrome types were compared. The patients were 32 years old (interquartile range, 27–42 years), including 196 (53.0%) females, with a median disease course of 6 months. Even though the infection was the most common (11.1%) inducement, the majority of the patients (78.6) had no obvious inducement. Hypertension was present in 136 (36.8%) patients at baseline. Microscopic hematuria was found in almost all patients, but only 66 (17.8%) patients showed macroscopic hematuria. The overall level of proteinuria was 1 (0.5–2.2) g/day; serum albumin was 40.3 (35.8–43.8) (g/L). The serum creatinine at the time of biopsy was 79 (59–108) *μ*mol/L, and eGFR was 97.2 (67.2–120.5) mL/min/1.73 m^2^. The scores of Oxford classification were as follows: M1 = 97.6%, E1 = 14.6%, S1 = 50.0%, and T1 = 52.2%/T2 = 18.4%.

### 3.2. Comparison of Clinicopathological Features among Different TCM Syndromes of Training Dataset

273 (73.8%) patients got QDSK, 66 (17.8%) patients got DBQY, and 31 (8.4%) patients were categorized as OTs. Although some difference was found in age (*P* = 0.025), the pairwise comparison did not reach a significant level. Any statistical difference was not observed in gender, disease course, and inducement among the three types of fundamental symptoms. The systolic blood pressure, diastolic blood pressure, and mean arterial pressure (MAP) of different types did not exhibit a significant difference; however, patients in OTs category showed a higher rate of hypertension than DBQY (*P* = 0.008). Although patients of QDSK seemed less likely to acquire macroscopic hematuria than OTs (*P* = 0.015) and some difference was seen in urinary red blood cell (*P* = 0.015), a pairwise test could not figure out the difference. Moreover, no obvious difference was observed in hemoglobin, proteinuria, uric acid, serum albumin, serum IgA, and C3 among those three types. For the renal function, the serum creatinine of patients with DBQY was <QDSK (*P* = 0.019) and OTs (*P* < 0.001). The eGFR of those with DBQY was > QDSK (*P* = 0.014) and OTs (*P* < 0.001), and the eGFR of OTs was <QDSK (*P* = 0.012). When dividing the patients' eGFR to different levels according to the 2012 Kidney Disease: Improving Global Outcomes (KDIGO) Clinical Practice Guideline for the Evaluation and Management of CKD [27], the levels of eGFR of OTs were lower than those with DBQY (*P* < 0.001) and QDSK (*P* = 0.005), primarily because the percentage of patients with an eGFR lower than 60 mL/min/1.73 m^2^ in OTs (41.9%) was significantly larger than that of those with DBQY (7.6%) and QDSK (20.5%). With respect to the pathological features, the three syndrome types seemed to have some differences in M scores (*P* = 0.032); however, the pairwise analysis did not show a sufficient difference, and a statistical difference was not observed in E, S, and T scores and the ratio of crescents.

### 3.3. Decision Trees

**Figure 1:**
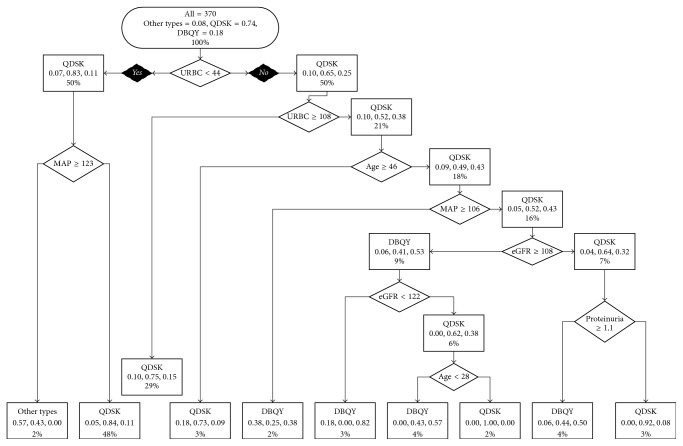
Decision tree model without MEST scores.

The decision tree without MEST scores for TCM syndromes is shown in Figure 1. The patients were classified according to the values of the variables at each node. Patients would fall into the nodes on the left if they fit the judgment in rhombuses. And patients would fall into the nodes on the right if they did not fit the judgment. The predicted syndrome types would be shown in the final nodes. For patients with a urinary red blood cell < 44/*μ*L at biopsy, the TCM fundamental syndrome was more likely to be QDSK; nevertheless, OTs would be more possible in those combining a MAP not less than 123 mmHg. For patients with urinary red blood cell not less than 108/*μ*L, the TCM syndrome was more likely to be QDSK. For patients with a urinary red blood cell between 44/*μ*L and 107/*μ*L, additional information of age, eGFR, MAP, and proteinuria was required for the classification of the syndromes.  Table 2 and Figure 1 summarized the predicting syndromes by the decision tree grouped by different recorded syndromes. The precision of QDSK was 93.4%, that of DBQY was 57.6%, and that of OTs was 22.6%. The decision tree model without MEST scores achieved total precision of 77.6%.

**Figure 2:**
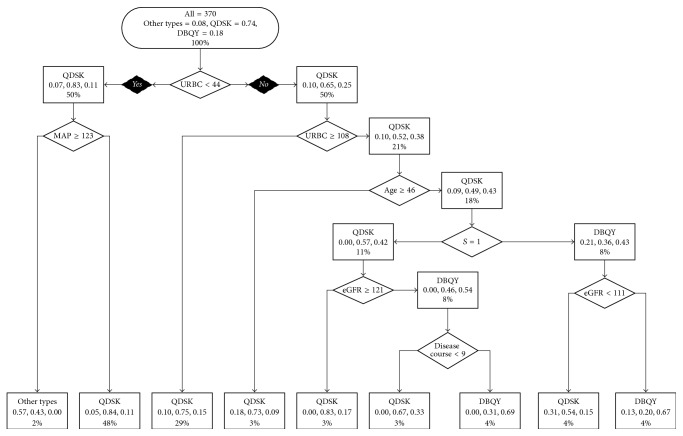
Decision tree model with MEST scores.

We also attempted to build a decision model with MEST scores (Figure 2). The tree was quite similar to the model described above but the condition of the judgment for patients below 46 years of age with a urinary red blood cell between 44/*μ*L and 107/*μ*L, S score of MEST, eGFR, and disease course was essential for decision-making. The predicting result of the model with MEST is presented in Table 3 and Figure 2. The precision of QDSK was 96.0%, that of DBQY was 31.8%, and that of OTs was 12.9%, rendering the total precision similar to the model without MEST scores.

### 3.4. Validation of Decision Trees

Data from 94 biopsy-proven IgA nephropathy adult patients from 1 January 2015 to 30 June 2016 in Guangdong Provincial Hospital of Chinese Medicine was collected to validate the models with or without MEST scores. 80 patients were differentiated as QDSK and 14 patients were differentiated as DBQY, and the clinicopathological parameters are shown in Table 4. The systolic blood pressure (*P* < 0.001), diastolic blood pressure (*P* = 0.001), and MAP (*P* < 0.001) of the validation dataset were larger compared to the training dataset (Table 5). The higher uric acid (*P* < 0.001) and serum creatinine (*P* < 0.001) and the lower eGFR (*P* < 0.001) implied that kidney injury was more severe in the validation dataset. However, the M score (*P* < 0.001) and T score (*P* < 0.001) were smaller in the validation dataset. We analyzed the eGFR of each T score (Table 6) and found that it declined from T0 and T1 to T2 in both training and validation datasets. In other words, the larger T score corresponded to less eGFR in both datasets. In the model without MEST (Table 7), 70 (87.5%) patients were accurately predicted as QDSK, leading to total precision of 75.5%. The total precision of the model with MEST was 80.9%, as 75 patients were accurately predicted as QDSK (Table 8). Because the precision of predicting DBQY was identical, additional indexes were evaluated to determine whether MEST scores supported the prediction of QDSK (Table 9). The PPV and NPV became larger after adding MEST scores to the clinical data. The values of IDI and NRI were > 0. Thus, adding MEST scores to the clinical data seemed to slightly improve the precision in predicting syndrome types.

## 4. Discussion

TCM syndromes of kidney diseases indeed have shortcomings in objectivity and consistency, leading to the difficulty in repeating syndrome classifications. But, in recent years, with the gradual deepening of TCM researches in the field of kidney diseases, some exact relationship between TCM syndromes and clinicopathological parameters was subsequently found. With appropriate TCM treatment based on syndrome differentiation, patients with kidney diseases can have their general condition and clinical indicators improved. Among the series of kidney diseases, IgA nephropathy is the most deeply explored one [28, 29]. A kidney disease research team led by academician explored the correlation between TCM syndromes and clinicopathological parameters among 1016 IgA nephropathy patients. They found that TCM syndromes were closely related to prognostic factors, like proteinuria, hypertension, and renal tissue injury [30, 31]. Under the guidance of the above studies, a randomized controlled trial was conducted, which found that treatments based on syndrome differentiation can decrease urine protein and serum creatinine without obvious adverse events [32, 33]. Soon after that, Chinese Association of Integrative Medicine issued the guideline to spread the evidence of treating IgA nephropathy based on syndrome differentiation. However, the diagnosis of a syndrome is largely dependent on the clinician's educational background and experience, and not all the renal physicians can master the skill in a short time. As the syndromes have such a close correlation with clinicopathological parameters, it is feasible to assist clinicians to classify TCM syndromes with clinicopathological parameters and help apply TCM treatments. The model could promote the precision of syndrome differentiation and make the syndromes more objective and repeatable. The promoted syndrome differentiation could benefit the consistency of the therapeutic effect of TCM as well as the performing of a large-scale prospective RCT in the future aiming to further verify the effect of TCM on IgA nephropathy. All these works will contribute to the spreading and development of Chinese Medicine.

In the present study, demographic data, clinicopathological parameters, and TCM fundamental syndromes of IgA nephropathy patients were retrospectively collected. The syndromes could be classified by decision tree models using five or six parameters. For those patients without biopsy, five variables were needed to classify the syndromes, including urinary red blood cell, MAP, age, eGFR, and proteinuria. For the biopsied patients, additional details were supplemented through the renal tissue. Then, the syndromes could be differentiated by urinary red blood cell, MAP, age, disease course, eGFR, and S score. Although the models with or without pathological features achieved identical precision in the training data, the model with MEST scores showed an advantage in validation, especially in predicting the syndrome types of QDSK.

When we compared the baseline clinicopathological features of the three syndrome types in training dataset, OTs performed poorly in multiple clinical indexes. OTs showed a higher proportion of hypertension than DBQY and were more likely to obtain macroscopic hematuria. In addition, relevant variables of renal function showed an extremely severe kidney injury in patients with OTs. As the OTs encompassed four syndrome types with small sample sizes, it was difficult to decipher the responsible factor; however, 27 (87.1%) patients had yin deficiency of liver and kidney or yang deficiency of spleen and kidney. A cross-sectional study surveyed 1148 Chinese IgA nephropathy patients' clinicopathological features [34]. The study found out that the renal function of those with yin deficiency of liver and kidney or yang deficiency of spleen and kidney was worse than those with DBQY or qi deficiency of lung and kidney. Moreover, the MAP of patients with yin deficiency of liver and kidney or yang deficiency of spleen and kidney was higher than that of those with DBQY or qi deficiency of lung and kidney. Therefore, the inferior value of OTs in multiple clinical indexes may be attributed to the types of yin deficiency of liver and kidney or yang deficiency of spleen and kidney.

To our knowledge, this is the first report differentiating TCM syndromes by building models with objective indexes in IgA nephropathy. In other diseases, some model has been developed to diagnose patients' syndromes [35–40]. Some of these studies were based on syndrome factors, which comprised subjective indexes, encountering challenges of extrapolation and repeatability [36, 40]. Cluster analysis can group the similar clinical features together, which may be related to different syndrome types; however, the similar features did not indicate cooccurrence of clinical features or symptoms [39]. The questionnaire is good for quantifying the variables, saving time and money, and enlarging the sample size rapidly [37]. Nevertheless, it is difficult to design the questionnaire so as to target reliability and validity among large crowds. The methods of classifying the TCM syndromes with objective variables were demonstrated by decision tree models using clinicopathological parameters of 370 IgA patients and then validating the models by 94 patients. The package of rpart is based on the method of Classification and Regression Trees [41] and classification or regression models of a very general structure using a two-stage procedure [42]. The resulting models of rpart have been represented as binary trees. Judgments of yes or no were the only requirement in the process of decision-making, which simplifies the interpretation. The decision-making processes are entirely visible, thus making it convenient to conduct validation, promotion, and application. The nodes of the trees are determined by the values of the variables, and the values of the cut-off points are based on the statistical probability. The variables that do not contribute to decision-making will be ignored. Unlike random forest, this method is less affected by partially missing data. Thus, the decision tree is sufficiently simple for use in clinical practice.

The classification and regression tree has several advantages of its own, irrespective of the management decision and statistical method. One obvious advantage is that the logic and operation are easily understood, making it intuitive and clear. The advantage is prominent when compared to the method with an opaque mechanism of weight and prediction, such as neural networks. Another advantage is the way of handling the missing data that can make full use of the data. The most common approach to handling missing values is deleting missing observations, but classification and regression tree only deletes the observations with missed dependent variables, while the observations with missed independent variables would be reserved. This method also exhibits adequate stability and versatility, because the dividing basis of variables is dependent on the rank of value instead of the numeric size, and thus even some outliers appearing in the dataset would have little influence on the result. The result is less affected by choice of variables than multiple regression analysis, as the addition and elimination of variables would not affect the result unless the variables were involved in the tree. Compared to the simple regressions, the classification and regression tree also eliminates the trouble in selecting variables, because, during the process of constructing the tree, the optimal cut-off point of each involved variable will be automatically selected.

Several limitations should be considered while interpreting our study. This is a retrospective study; thus, the types of TCM syndromes were not strictly classified by identical criteria, and necessary postreclassification was performed. Except for the combined type of OTs, only 2 syndromes, QDSK and DBQY, entered into the statistical analysis, making the generalization of the findings to other TCM syndromes uncertain. The precision of classifying OTs was much less than 1/3, which might be due to the type mixed with several types, and the characteristic of each original type was confused. The clinicopathological parameters of training and validation datasets were quite different, especially in renal function and pathological features. The limited sample size limitedly represented the population of all IgA nephropathy patients.

In conclusion, the decision trees on the basis of clinical indexes can help classify the types of TCM syndromes, and the pathological features may slightly improve the precision. Similar studies based on larger sample size or prospective studies would be useful for further exploration.

## Figures and Tables

**Table 1:** Comparison of demographic and clinicopathological parameters among different TCM syndromes of training dataset.

	Total (*n* = 370)	Qi deficiency of spleen and kidney (*n* = 273)	Deficiency of both qi and yin (*n* = 66)	Other types (*n* = 31)	*P* value
Age (years)	32 (27–42)	33 (27–42)	29 (25–36)	37 (28–46)	0.025^a^
Female, *n* (%)	196 (53.0)	139 (50.9)	42 (63.6)	15 (48.4)	0.154
Disease course (months)	6 (2–23)	5 (1–23)	12 (3–25)	6 (2–13)	0.186
*Inducement, n (%)*					
Infection	41 (11.1)	31 (11.4)	5 (7.6)	5 (16.1)	0.780
Fatigue	13 (3.5)	11 (4.0)	1 (1.5)	1 (3.2)	
Pregnancy	25 (6.8)	19 (7.0)	5 (7.6)	1 (3.2)	
No obvious inducement	291 (78.6)	212 (77.7)	55 (83.3)	24 (77.4)	
Systolic BP (mmHg)	120 (110–132)	120 (110–132)	120 (108–130)	125 (118–146)	0.070
Diastolic BP (mmHg)	80 (70–86)	80 (70–89)	79 (70–80)	80 (72–100)	0.097
MAP (mmHg)	93 (86–101)	93 (86–101)	91 (82–97)	94 (88–113)	0.069
Hypertension, *n* (%)	136 (36.8)	101 (37.0)	18 (27.3)	17 (54.8)	0.031^b^
Macroscopic hematuria, *n* (%)	66 (17.8)	39 (14.3)	17 (25.8)	10 (32.3)	0.008^c^
U-RBC (/*μ*L)	45 (15–136)	32 (11–134)	60 (30–110)	74 (25–179)	0.015^d^
Hemoglobin (g/L)	128.2 ± 20.1	129.5 ± 20.2	126.1 ± 16.2	121.1 ± 25.2	0.055
Proteinuria (g/day)	1.0 (0.5–2.2)	1.0 (0.5–2.1)	1.1 (0.5–2.5)	1.1 (0.5–2.7)	0.766
Uric acid (*μ*mol/L)	340 (274–429)	344 (277–433)	316 (255–408)	358 (302–460)	0.170
Serum albumin (g/L)	40.3 (35.8–43.8)	40.6 (36.1–44.0)	40.2 (36.4–42.8)	39.2 (34.2–42.1)	0.405
Serum creatinine (*μ*mol/L)	79 (59–108)	80 (61–107)	67 (56–88)	123 (63–183)	0.001^e^
eGFR (mL/min/1.73 m^2^)	97.2 (67.2–120.5)	97.1 (66.3–119.9)	115.3 (82.0–125.7)	70.6 (27.7–114.2)	<0.001^f^
*eGFR (mL/min/1.73 m* ^*2*^)					
<15	6 (1.6)	3 (1.1)	0	3 (9.7)	0.001^g^
>15, ≤30	15 (4.1)	9 (3.3)	1 (1.5)	5 (16.1)	
>30, ≤60	53 (14.3)	44 (16.1)	4 (6.1)	5 (16.1)	
>60, ≤90	94 (25.4)	67 (24.5)	19 (28.8)	8 (25.8)	
>90	202 (54.6)	150 (54.9)	42 (63.6)	10 (32.3)	
Serum IgA (g/L)	2.85 (2.25–3.58)	2.84 (2.28–3.58)	2.81 (2.19–3.39)	2.97 (2.51–4.02)	0.246
Serum C3 (mg/L)	0.98 (0.87–1.11)	0.98 (0.87–1.12)	0.98 (0.85–1.10)	0.96 (0.85–1.08)	0.611
*Oxford MEST scores*					
M1, *n* (%)	361 (97.6)	269 (98.5)	64 (97.0)	28 (90.3)	0.032^h^
E1, *n* (%)	54 (14.6)	41 (15.0)	11 (16.7)	2 (6.5)	0.384
S1, *n* (%)	185 (50.0)	142 (52.0)	32 (48.5)	11 (35.5)	0.210
T1, *n* (%)	193 (52.2)	146 (53.5)	30 (45.5)	17 (54.8)	0.242
T2, *n* (%)	68 (18.4)	48 (17.6)	12 (18.2)	8 (25.8)	

BP, blood pressure; MAP, mean arterial pressure; U-RBC, urinary red blood cell; eGFR, estimated glomerular filtration rate; QDSK, qi deficiency of spleen and kidney; DBQY, deficiency of both qi and yin; OTs, other types.

^a^ *P*_QDSK  versus  DBQY_ = 0.051, *P*_QDSK  versus  OTs_ = 0.999, and *P*_DBQY  versus  OTs_ = 0.059.

^b^ *P*_QDSK  versus  DBQY_ = 0.137, *P*_QDSK  versus  OTs_ = 0.053, and *P*_DBQY  versus  OTs_ = 0.008; *α*′ = (*α*/3) = 0.05/3 = 0.017.

^c^ *P*_QDSK  versus  DBQY_ = 0.028, *P*_QDSK  versus  OTs_ = 0.015, and *P*_DBQY  versus  OTs_ = 0.628; *α*′ = (*α*/3) = 0.05/3 = 0.017.

^d^ *P*_QDSK  versus  DBQY_ = 0.053, *P*_QDSK  versus  OTs_ = 0.139, and *P*_DBQY  versus  OTs_ = 0.999.

^e^ *P*_QDSK  versus  DBQY_ = 0.019, *P*_QDSK  versus  OTs_ = 0.054, and *P*_DBQY  versus  OTs_ < 0.001.

^f^ *P*_QDSK  versus  DBQY_ = 0.014, *P*_QDSK  versus  OTs_ = 0.012, and *P*_DBQY  versus  OTs_ < 0.001.

^g^ *P*_QDSK  versus  DBQY_ = 0.231, *P*_QDSK  versus  OTs_ = 0.005, and *P*_DBQY  versus  OTs_ < 0.001.

^h^ *P*_QDSK  versus  DBQY_ = 0.601, *P*_QDSK  versus  OTs_ = 0.025, and *P*_DBQY  versus  OTs_ = 0.323; *α*′ = (*α*/3) = 0.05/3 = 0.017.

**Table 2:** Patients' distribution of predicting syndromes based on the decision tree without MEST scores.

Predicted syndromes	Recorded syndromes		
	Qi deficiency of spleen and kidney (*n* = 273)	Deficiency of both qi and yin (*n* = 66)	Other types (*n* = 31)
Qi deficiency of spleen and kidney	255 (93.4)	38 (57.6)	21 (67.7)
Deficiency of both qi and yin	13 (4.8)	25 (37.9)	3 (9.7)
Other types	5 (1.8)	3 (4.5)	7 (22.6)

Note: the accuracy of the model was 77.6%.

**Table 3:** Patients' distribution of predicting syndromes based on the decision tree with MEST scores.

Predicted syndromes	Recorded syndromes		
	Qi deficiency of spleen and kidney (*n* = 273)	Deficiency of both qi and yin (*n* = 66)	Other types (*n* = 31)
Qi deficiency of spleen and kidney	262 (96.0)	45 (68.2)	25 (80.6)
Deficiency of both qi and yin	8 (2.9)	21 (31.8)	2 (6.5)
Other types	3 (1.1)	0	4 (12.9)

Note: the accuracy of the model was 77.6%.

**Table 4:** Comparison of demographic and clinicopathological parameters among different TCM syndromes of validation dataset.

	Total (*n* = 94)	Qi deficiency of spleen and kidney (*n* = 80)	Deficiency of both qi and yin (*n* = 14)	*P* value
Age (years)	31 (25–42)	33 (27–42)	27 (22–34)	0.015
Female, *n* (%)	47 (50.0)	40 (50.0)	7 (50.0)	0.999
Disease course (months)	9 (1–21)	12 (2–24)	1.5 (1–7.5)	0.018
*Inducement, n (%)*				
Infection	16 (17.0)	12 (15.0)	4 (28.6)	0.327
Fatigue	3 (3.2)	2 (2.5)	1 (7.1)	
Pregnancy	1 (1.1)	1 (1.2)	0	
No obvious inducement	74 (78.7)	65 (81.2)	9 (64.3)	
Systolic BP (mmHg)	127 (117–143)	129 (117–143)	122 (116–132)	0.183
Diastolic BP (mmHg)	84 (75–93)	85 (76–94)	81 (72–85)	0.056
MAP (mmHg)	98 (90–107)	100 (91–110)	93 (87–99)	0.079
Hypertension, *n* (%)	39 (41.5)	36 (45.0)	3 (21.4)	0.099
Macroscopic hematuria, *n* (%)	9 (9.6)	8 (10.0)	1 (7.1)	0.999
U-RBC (/*μ*L)	40 (13–172)	41 (18–172)	23 (3–122)	0.128
Hemoglobin (g/L)	128.2 ± 20.1	124.7 ± 21.4	141.9 ± 21.2	0.006
Proteinuria (g/day)	1.0 (0.5–2.4)	1.0 (0.5–2.4)	0.8 (0.4–1.8)	0.644
Uric acid (*μ*mol/L)	410 (328–519)	410 (335–521)	400 (306–489)	0.425
Serum albumin (g/L)	39.4 (35.3–42.9)	38.6 (34.5–42.2)	42.9 (36.9–45.2)	0.061
Serum creatinine (*μ*mol/L)	96 (72–151)	104 (78–154)	72 (64–95)	0.012
eGFR (mL/min/1.73 m^2^)	78.8 (50.5–109.3)	73.3 (46.6–95.8)	109.8 (90.8–123.8)	0.002
*eGFR (mL/min/1.73 m* ^*2*^)				
<15	5 (5.3)	5 (6.2)	0	0.006
>15, ≤30	9 (9.6)	8 (10.0)	1 (7.1)	
>30, ≤60	20 (21.3)	19 (23.8)	1 (7.1)	
>60, ≤90	22 (23.4)	21 (26.2)	1 (7.1)	
>90	38 (40.4)	27 (33.8)	11 (78.6)	
Serum IgA (g/L)	2.99 (2.23–3.57)	2.98 (2.22–3.62)	2.99 (2.23–3.45)	0.866
Serum C3 (mg/L)	1.05 (0.87–1.20)	1.04 (0.85–1.16)	1.20 (1.08–1.26)	0.005
*Oxford MEST scores*				
M1, *n* (%)	79 (86.8)	69 (89.6)	10 (71.4)	0.085
E1, *n* (%)	11 (12.1)	10 (13.0)	1 (7.1)	0.690
S1, *n* (%)	55 (60.4)	48 (62.3)	7 (50.0)	0.554
T1, *n* (%)	23 (25.3)	22 (28.6)	1 (7.1)	0.133
T2, *n* (%)	16 (17.6)	14 (18.2)	2 (14.3)	

BP, blood pressure; MAP, mean arterial pressure; U-RBC, urinary red blood cell; eGFR, estimated glomerular filtration rate.

**Table 5:** Comparison of demographic and clinicopathological parameters between training and validation datasets.

	Training dataset (*n* = 370)	Validation dataset (*n* = 94)	*P* value
Age (years)	32 (27–42)	31 (25–42)	0.899
Female, *n* (%)	196 (53.0)	47 (50.0)	0.606
Disease course (months)	6 (2–23)	9 (1–21)	0.946
*Inducement, n (%)*			
Infection	41 (11.1)	16 (17.0)	0.089
Fatigue	13 (3.5)	3 (3.2)	
Pregnancy	25 (6.8)	1 (1.1)	
No obvious inducement	291 (78.6)	74 (78.7)	
Systolic BP (mmHg)	120 (110–132)	127 (117–143)	<0.001
Diastolic BP (mmHg)	80 (70–86)	84 (75–93)	0.001
MAP (mmHg)	93 (86–101)	98 (90–107)	<0.001
Hypertension, *n* (%)	136 (36.8)	39 (41.5)	0.285
Macroscopic hematuria, *n* (%)	66 (17.8)	9 (9.6)	0.052
U-RBC (/*μ*L)	45 (15–136)	40 (13–172)	0.798
Hemoglobin (g/L)	128.2 ± 20.1	128.2 ± 20.1	0.693
Proteinuria (g/day)	1.0 (0.5–2.2)	1.0 (0.5–2.4)	0.764
Uric acid (*μ*mol/L)	340 (274–429)	410 (328–519)	<0.001
Serum albumin (g/L)	40.3 (35.8–43.8)	39.4 (35.3–42.9)	0.238
Serum creatinine (*μ*mol/L)	79 (59–108)	96 (72–151)	<0.001
eGFR (mL/min/1.73 m^2^)	97.2 (67.2–120.5)	78.8 (50.5–109.3)	<0.001
*eGFR (mL/min/1.73 m* ^*2*^)			
<15	6 (1.6)	5 (5.3)	0.001
>15, ≤30	15 (4.1)	9 (9.6)	
>30, ≤60	53 (14.3)	20 (21.3)	
>60, ≤90	94 (25.4)	22 (23.4)	
>90	202 (54.6)	38 (40.4)	
Serum IgA (g/L)	2.85 (2.25–3.58)	2.99 (2.23–3.57)	0.736
Serum C3 (mg/L)	0.98 (0.87–1.11)	1.05 (0.87–1.20)	0.045
*Oxford MEST scores*			
M1, *n* (%)	361 (97.6)	79 (86.8)	<0.001
E1, *n* (%)	54 (14.6)	11 (12.1)	0.538
S1, *n* (%)	185 (50.0)	55 (60.4)	0.074
T1, *n* (%)	193 (52.2)	23 (25.3)	<0.001
T2, *n* (%)	68 (18.4)	16 (17.6)	

BP, blood pressure; MAP, mean arterial pressure; U-RBC, urinary red blood cell; eGFR, estimated glomerular filtration rate.

**Table 6:** The eGFR of different T scores in training and validation datasets.

	Training dataset	Validation dataset
T0	119.1 (102.7–126.1)	94.8 (78.4–118.8)
T1	94.1 (71.5–118.8)	57.3 (38.9–89.3)
T2	53.4 (35.3–72.4)	27.6 (17.3–47.9)

Note: the unit used in the form was mL/min/1.73 m^2^.

**Table 7:** Validating the decision tree without MEST scores.

Predicted syndromes	Recorded syndromes	
	Qi deficiency of spleen and kidney (*n* = 80)	Deficiency of both qi and yin (*n* = 14)
Qi deficiency of spleen and kidney	70 (87.5)	13 (92.9)
Deficiency of both qi and yin	4 (5.0)	1 (7.1)
Other types	6 (7.5)	0

Note: the accuracy of the model was 75.5%.

**Table 8:** Validating the decision tree with MEST scores.

Predicted syndromes	Recorded syndromes	
	Qi deficiency of spleen and kidney (*n* = 80)	Deficiency of both qi and yin (*n* = 14)
Qi deficiency of spleen and kidney	75 (93.75)	13 (92.9)
Deficiency of both qi and yin	0	1 (7.1)
Other types	5 (6.25)	0

Note: the accuracy of the model was 80.9%.

**Table 9:** Discrimination in performance of different models predicting syndrome of qi deficiency of spleen and kidney.

Model without MEST					Model with MEST					IDI	NRI
Prediction	QDSK (*n* = 80)	nQDSK (*n* = 14)	PPV	NPV	Prediction	QDSK (*n* = 80)	nQDSK (*n* = 14)	PPV	NPV		
QDSK	70	13	0.84	0.09	QDSK	75	13	0.85	0.17	0.08	0.13
nQDSK	10	1			nQDSK	5	1				

Note: QDSK, qi deficiency of spleen and kidney; nQDSK, no qi deficiency of spleen and kidney; PPV, positive predictive value; NPV, negative predictive value; IDI, integrated discrimination improvement; NRI, net reclassification index.
